# Effect of lactobacilliprobiotic strains on IFN- γ, IL-2 and TNF-α- production in the peripheral blood cells culture of clinically healthy donors

**DOI:** 10.1186/1878-5085-5-S1-A135

**Published:** 2014-02-11

**Authors:** Viktoria V Mokrozub, Lidia P Babenko, Olena Yu Sokolvyak, Liudmyla M Lazarenko, Mykola Ya Spivak

**Affiliations:** 1 D.K. Zabolotny Institute of microbiology and virology, National Academy of Sciences of Ukraine, 154, Zabolotny st., 03680, Kyiv, Ukraine

## Introduction

The concept of using *Lactobacillus* species for disease treatment and prevention as well as health restoration and maintenance is not new. Probiotics have been used therapeutically to modulate immunity, lower cholesterol, treat rheumatoid arthritis, prevent cancer, improve lactose intolerance, and prevent or reduce the effects of atopic dermatitis, Crohn’s disease, diarrhea, and constipation as well as candidiasis and urinary tract infections.

## Aim

was study the ability of *L. acidophilus* IMV B-7279 and *L. delbrueckii* subsp. *bulgaricus* IMV B-7281 probiotic strains to influence on Th1-type cytokines production.

## Methods

Experimental studies were performed on blood of clinically healthy donors (females, 26 year old). Venous blood was diluted in a ratio 1:4 of cell medium RPMI-1640. Cytokines were induced in diluted blood 500 µl via PHA 500 µl (40 µg/ml). Ability of peripheral blood cells to produce IFN-γ and IL-2 was studied via added probiotic strains (5 × 10^9^ cells/ml) into diluted blood. Cytokines level were investigated in cells supernatants via IFA-method.

## Results

Thus, the ability of peripheral blood cells to produce IFN-γ and IL-2 was increased under the influence of *L. delbrueckii* subsp. *bulgaricus* IMV B-7281. The concentration of IFN-γ in the culture medium increased to 322.4 ± 7.5 pg/ml against 123.2 ± 13.8 pg/ml (*p* < 0.05) in control; IL-2 – 39.3 ± 1.1 pg/ml (10.4 ± 1.7 pg/ml in control; *p* < 0.05). *L. acidophilus* IMV B-7279 had no activating effect on the production of these cytokines by means of peripheral blood cells. The concentration of IFN-γ in the culture medium was 147.3 ± 15.9 pg/ml (*p*> 0.05), and IL-2 was at the control level (11.5 ± 1.3 pg/ml; *p*> 0.05).

## Conclusion

The results of our study suggested that *L. acidophilus* IMV B-7279 and *L. delbrueckii* subsp. *bulgaricus* IMV B-7281 probiotic strains have no activating effect on production of TNF-α. The concentration of this cytokine in the culture medium of peripheral blood cells, which were treated with *L. acidophilus* IMV B-7279 or *L. delbrueckii* subsp. *bulgaricus* IMV B-7281, amounted to 8.7 ± 0.1 and 8.9 ± 0.3 pg/ml against 8.5 ± 0.5 pg/ml in control (*p* > 0.05). *L. delbrueckii* subsp. *bulgaricus* IMV B-7281 probiotic strain induced *in vitro* production of Th1-type cytokines in the culture of peripheral blood cells.

## Recommendations

Therefore, this strain is prospective for correction of immune response in infectious and other diseases with clinically unfavorable course accompanied by inhibition of Th1-type T-helpers/inductors.

